# A brainnetome atlas-based methamphetamine dependence identification using neighborhood component analysis and machine learning on functional MRI data

**DOI:** 10.3389/fncel.2022.958437

**Published:** 2022-09-27

**Authors:** Yanan Zhou, Jingsong Tang, Yunkai Sun, Winson Fu Zun Yang, Yuejiao Ma, Qiuxia Wu, Shubao Chen, Qianjin Wang, Yuzhu Hao, Yunfei Wang, Manyun Li, Tieqiao Liu, Yanhui Liao

**Affiliations:** ^1^Department of Psychiatry, National Clinical Research Center for Mental Disorders, The Second Xiangya Hospital of Central South University, Changsha, China; ^2^Department of Psychiatry, Brain Hospital of Hunan Province (The Second People's Hospital of Hunan Province), Changsha, China; ^3^Department of Psychiatry, School of Medicine, Sir Run-Run Shaw Hospital, Zhejiang University, Hangzhou, China; ^4^Department of Psychological Sciences, College of Arts and Sciences, Texas Tech University, Lubbock, TX, United States

**Keywords:** methamphetamine, machine learning, brainnetome atlas, neighborhood component analysis, resting-state functional magnetic resonance imaging, classification

## Abstract

Addiction to methamphetamine (MA) is a major public health concern. Developing a predictive model that can classify and characterize the brain-based biomarkers predicting MA addicts may directly lead to improved treatment outcomes. In the current study, we applied the support vector machine (SVM)-based classification method to resting-state functional magnetic resonance imaging (rs-fMRI) data obtained from individuals with methamphetamine use disorder (MUD) and healthy controls (HCs) to identify brain-based features predictive of MUD. Brain connectivity analyses were conducted for 36 individuals with MUD as well as 37 HCs based on the brainnetome atlas, and the neighborhood component analysis was applied for feature selection. Eighteen most relevant features were screened out and fed into the SVM to classify the data. The classifier was able to differentiate individuals with MUD from HCs with a high prediction accuracy, sensitivity, specificity, and AUC of 88.00, 86.84, 89.19, and 0.94, respectively. The top six discriminative features associated with changes in the functional activity of key nodes in the default mode network (DMN), all the remaining discriminative features are related to the thalamic connections within the cortico-striato-thalamo-cortical (CSTC) loop. In addition, the functional connectivity (FC) between the bilateral inferior parietal lobule (IPL) and right cingulate gyrus (CG) was significantly correlated with the duration of methamphetamine use. The results of this study not only indicated that MUD-related FC alterations were predictive of group membership, but also suggested that machine learning techniques could be used for the identification of MUD-related imaging biomarkers.

## Introduction

Methamphetamine (MA) is an illegal psychoactive drug that is highly addictive and widely abused (Qie et al., 2017). Prolonged MA use can eventually lead to drug addiction and cause serious damage to multiple organ systems, especially the central nervous system (Prakash et al., 2017). It interacts with nervous systems to modulate drug-related circuitry, resulting cognitive impairment, memory loss, motor skill impairment, attention deficit, psychotic disorders, violent behavior, etc. (Rusyniak, 2011), which is closely related to MA-induced neurotoxicity and neuroinflammation (Kim et al., 2020). Despite these serious consequences, its use has grown significantly not only in China (China Drug Situation Report, 2020) but in many other countries worldwide in recent years (Bach et al., 2020). Furthermore, the treatment outcome for MA addiction is poor, and the relapse rate remains high after remission (Gouzoulis-Mayfrank et al., 2017), one of the reasons is the lack of clinically objective addiction diseases biomarkers that, if available, may directly lead to improved treatment outcomes.

Neuroimaging can be a powerful tool for identifying the possible relevant imaging biomarkers. Of late, resting-state functional magnetic resonance imaging (rs-fMRI), which measures hemodynamic changes in the blood-oxygen-level-dependent (BOLD) signals caused by spontaneous neural activity (Lee et al., 2013), has been explored to examine underlying neurobiological mechanisms of addiction including MA. In this approach, differences in neural function can be identified by measuring the connectivity from the BOLD time series between the various regions of the brain. By using rs-fMRI data, previous studies examined the effects of MA on the functional connectivity (FC) of the brain and discovered significant differences that may be related to some of the core symptoms observed in MA addicts (Kohno et al., 2014; Dean et al., 2015; Zhang et al., 2018; Li et al., 2020). Although these findings provide new insights into the neural features of MA addiction, the great majority of these research are based on explanatory models and the generalizability of the results remains unclear. Importantly, with rare exceptions (Yan et al., 2021), most of these studies are restricted to using conventional univariate analysis (such as analysis of variance and two-sample *T*-test) to identify differences in neural processing between MA addicts and HCs, overlooking multivariate patterns in the rs-fMRI data. This type of analysis is not very beneficial for revealing differences in the whole spatial pattern of brain changes between groups or for identifying patients at the individual level, which limits its applicability in clinical diagnoses and treatment. Hence, a method more suitable for the above circumstances is needed.

Machine learning (ML) can address the above limitations. In contrast to conventional univariate methods, ML-based pattern classification is a kind of multivariate analyses that train classifiers to decode behaviors, mental states, stimuli, and other variables of interest from rs-fMRI data and thereby showing the data contain information about them (Pereira et al., 2009). Further, it trains computers to iteratively improve their performance in identifying relationships between variables and produce more superior predictive models (Cortes and Vapnik, 1995). ML-based approaches have been widely applied with rs-fMRI data in studies on a variety of brain disorders such as schizophrenia (Cai et al., 2020; Steardo et al., 2020), depression (Gao et al., 2018; Han et al., 2019), Alzheimer's (Castellazzi et al., 2020) and Parkinson's disease (Gu et al., 2016; Rubbert et al., 2019), nicotine (Pariyadath et al., 2014; Wetherill et al., 2019), cocaine (Yip et al., 2019), or MA addiction (Li et al., 2019; Ding et al., 2020). Among these approaches, support vector machines (SVM) is the most commonly used one due to its better prediction accuracy and lower sensitivity to noise when handling multidimensional data (Craddock et al., 2009). SVM has been shown to be successful in identifying neural activation patterns (Haxby, 2012) as well as individual-level differences (Shen et al., 2020) from rs-fMRI data. For example, Song et al. found that the default-mode network is the most informative network in predicting internet gaming disorder based on SVM classification with rs-fMRI data (Song et al., 2021). However, to date, very few rs-fMRI data has been explored with the use of SVM in MA addicts.

Thus, in the present study, we used an SVM-based ML approach to analyze rs-fMRI data, looking to establish an effective model to differentiate MA addicts from HCs and to identify the most powerful discriminant features for the classification. We also investigated the relationship between discriminant features and MA use behaviors using spearman correlation analysis. Hopefully, this study will improve our understanding of the underlying pathological mechanisms of MA addiction through multivariate approaches.

## Materials and methods

### Participants

All participants provided voluntary written informed consent after being fully informed of the study procedures. This study was approved by the Ethics Committee of the Second Xiangya Hospital of Central South University (No. S095, 2013).

Forty-six inpatients with MA use disorders (MUD) were recruited between August 2019 and November 2019 from the Kangda Voluntary Drug Rehabilitation Center located in Changsha, China. The inclusion criteria for the MUD group were: (a) Han males aged 18–45; (b) having completed at least 6 years of formal education; (c) fluent in Chinese and able to understand instructions; (d) diagnosed with MUD by at least two certified psychiatrists according to the Diagnostic and Statistical Manual of Mental Disorders, Fifth Edition (DSM-5); and (e) positive for MA and negative for other drugs in the urine toxicology test on admission to the hospital. Individuals with a history of major chronic medical illnesses, neurological diseases, or mental disorders before MA use were excluded. Individuals with contraindications for MRI scanning (e.g., claustrophobia and implantation of metallic or electronic devices) were also excluded. Forty-five HCs were recruited between September 2019 and January 2020 from local communities *via* social media and online advertisements with the same inclusion and exclusion criteria for the MUD group, plus that they needed to have no history of drug abuse or dependence (except for nicotine).

### Clinical assessments

After the initial on-site screening, a detailed clinical interview was conducted by two trained psychiatrists before the fMRI scanning was performed. For the MUD group, the clinical interview and fMRI scanning were conducted when participants had no significant withdrawal symptoms. Information on the demographics (e.g., age, educational attainment) and MA measures (e.g., duration of MA use, average frequency of MA use in the past year before abstinence, average frequency of MA use in the past month before abstinence, average dose of MA use in typical occasion, and withdrawal time) were gathered by self-designed questionnaires.

### MR imaging acquisition

The MRI scanning was performed at the Magnetic Resonance Imaging Center of Hunan Children's Hospital, Changsha, China. The scanning was performed using a 3.0 T Siemens Skyra Munich MRI system equipped with a 16-channel head coil. For the resting-state scanning, participants were asked to remain awake while keeping their eyes closed.

A gradient echo-planar imaging sequence was applied to obtain resting state imaging data with the following parameters: TR = 2,000 ms, TE = 30 ms, flip angle = 78°, number of slices = 33, slice thickness = 3.5 mm, slice gap = 0.7 mm, field of view = 224 × 224 mm, and voxel size = 3.5 × 3.5 × 3.5 mm^3^. A 3D magnetization preparing rapid acquisition gradient echo sequence (3D MPRAGE) was applied to obtain T1-weighted images with the following parameters: TR = 2,530 ms, TE = 2.98 ms, flip angle = 7°, number of slices = 176, slice thickness = 1 mm, slice gap = 0 mm, field of view = 256 mm × 256 mm, and voxel size = 1 × 1 × 1 mm^3^.

### Preprocessing of MR images

The rs-fMRI images were preprocessed by using DPARSF (version 4.1) through the following steps: (1) discard the first 10 volumes, considering the magnetic saturation and adaptation of the participants to the circumstances; (2) proofread slice-timing with Fourier interpolation followed by removal of physiological artifact; (3) perform head motion correction to exclude participants with excessive head movement. For quality control, participants with head motion >2 mm or rotation > 2° were eliminated; (4) co-registration of rs-fMRI images to subject-specific T1 structural image; (5) spatial normalization to the Montreal Neurological Institute (MNI) standard space by resampling to 3 mm^3^; (6) spatial smoothing with a full-width-at-half-maximum (FWHM). Gaussian kernel of 4 mm to improve signal detection; (7) remove low-frequency fluctuations and high-frequency noise by using a band-pass temporal filter (0.01–0.1 Hz); (8) regress out nuisance signals, such as global signal, white matter signal, cerebrospinal fluid signal, and the Friston 24 head motion parameters.

### Brainnetome atlas-based FC

To extract functional regions, we used the newly developed human brainnetome atlas as an initial segmentation of the brain into 246 subregions. The human brainnetome atlas is a precise connectivity-based parcellation atlas that is fine-grained and cross-validated, containing both anatomical and functional information and providing mapping between the delineated structures and mental processes (visit http://atlas.brainnetome.org/ for more details) (Fan et al., 2016). In this study, each brain subregion was considered as a node of the functional brain network. The time course of each node was averaged, and Pearson correlation coefficients between the connected nodes were identified as the edge weight of the functional network (Pedersen et al., 2018), thus generating a 246 × 246 whole-brain FC matrix for each subject. Then, the matrices underwent conversion through Fisher's r-to-Z transformation into binary, undirected connection matrices.

### Feature selection

Feature selection was performed using neighborhood component analysis (NCA), a relatively new and less known method for feature selection, for the learning of the Mahalanobis distance metric in the k-nearest neighbor classification algorithm (Goldberger et al., 2005). It facilitated selection of features as it does not assume any parametric distribution of the features; it is also suitable for multiclass classification using high dimensional features (Yang et al., 2012). Thus, this method is a feature weighting scheme to select best feature subsets based on the weights while minimizing the cross-validation error of the training data. The features assigned with non-zero weights were then retained and fed into a linear SVM to classify the data.

### Support vector machine classifier

Support vector machine is the most popular algorithm for classification among ML techniques (Luts et al., 2010). For example, using a set of features and labels, we trained the SVM based on the training dataset, which mapped the set of features to their respective labels. During the training process, the optimum hyperplane that separated the training data by the maximum margin was found. Thus, with a new dataset of features derived from observation, we could then utilize the SVM to predict a label for this new observation. In this study, SVM was implemented in the LIBSVM classification library. We assessed the classification performance in a framework of permutation tests. Using the actual value of classification accuracy after the SVM analysis as the statistic, permutation tests were performed to estimate the statistical significance of the value. Specifically, the class labels of the training data were randomly changed beforehand, and leave-one-out cross-validation was then performed on the permuted dataset. This permutation process was repeated 10,000 times. The classification performance was regarded as reliable when the generalization rate obtained by the classifier trained on the real class labels exceeded the 95% confidence interval of the classifier trained on randomly relabeled class labels. The accuracy, sensitivity, and specificity were calculated to quantify the prediction performance of the classifiers. The area under the receiver operating characteristics curves (AUC) was also calculated to quantify the classification power, with a greater AUC indicating a higher classification power.

### Statistical analysis

The differences in demographic characteristics between the MUD group and HCs were tested with the independent-sample *T*-test using SPSS 23.0 software. Furthermore, Spearman correlation analysis was performed to explore the behavioral significance of alternative FC network in the MUD patients. To be specific, we explored the correlations between the FC fed into the SVM as features and MA measures (duration of MA use, average frequency in the past year, average frequency in the past month, average dose of MA use, withdrawal time). The statistical significance level was set at *p* < 0.05.

## Results

### Demographics and clinical data

After excluding those who had incomplete or abnormal scan, or excessive head motion in the process of image preprocessing, the final dataset comprised 36 MUD and 37 HCs participants for analyses. The demographics and clinical characteristics are summarized in Table 1. The MUD group was older (31.06 ± 5.60) and lower educated (11.42 ± 3.15) to those in the HC group (age, 26.35 ± 7.13; education, 13.84 ± 3.38 years). The duration of self-reported MA use was 6.17 ± 3.34 years, average dose of MA use in typical occasion was 0.36 ± 0.21 g, withdrawal time was 63.83 ± 43.23 days. The average frequency of MA use in the past month before abstinence was significantly higher than that in the past year before abstinence.

**Table 1:** Participant demographics and clinical characteristics.

**Variables**	**MUD (*n* = 36)**	**HCs (*n* = 37)**	***p*** **-Value**
Age	31.06 (5.60)	26.35 (7.13)	<0.001
Education years	11.42 (3.15)	13.84 (3.38)	<0.001
Duration of MA use (years)	6.17 (3.34)	-	-
Average dose of MA use (g)	0.36 (0.21)	-	-
Withdrawal time (days)	63.83 (43.23)	-	-
Frequency in the past year		-	-
At least once per day	4 (11.1%)	-	-
Once every 2–3 days	9 (25.0%)	-	-
Once every 4–9 days	11 (30.6%)	-	-
Once every 10 days or more	12 (33.3%)	-	-
Frequency in the past month		-	-
At least once per day	6 (16.7%)	-	-
Once every 2–3 days	12 (33.3%)	-	-
Once every 4–9 days	6 (16.7%)	-	-
Once every 10 days or more	12 (33.3%)	-	-

MUD, methamphetamine use disorder; HCs, Health controls.

### Discriminative features

**Figure 1:**
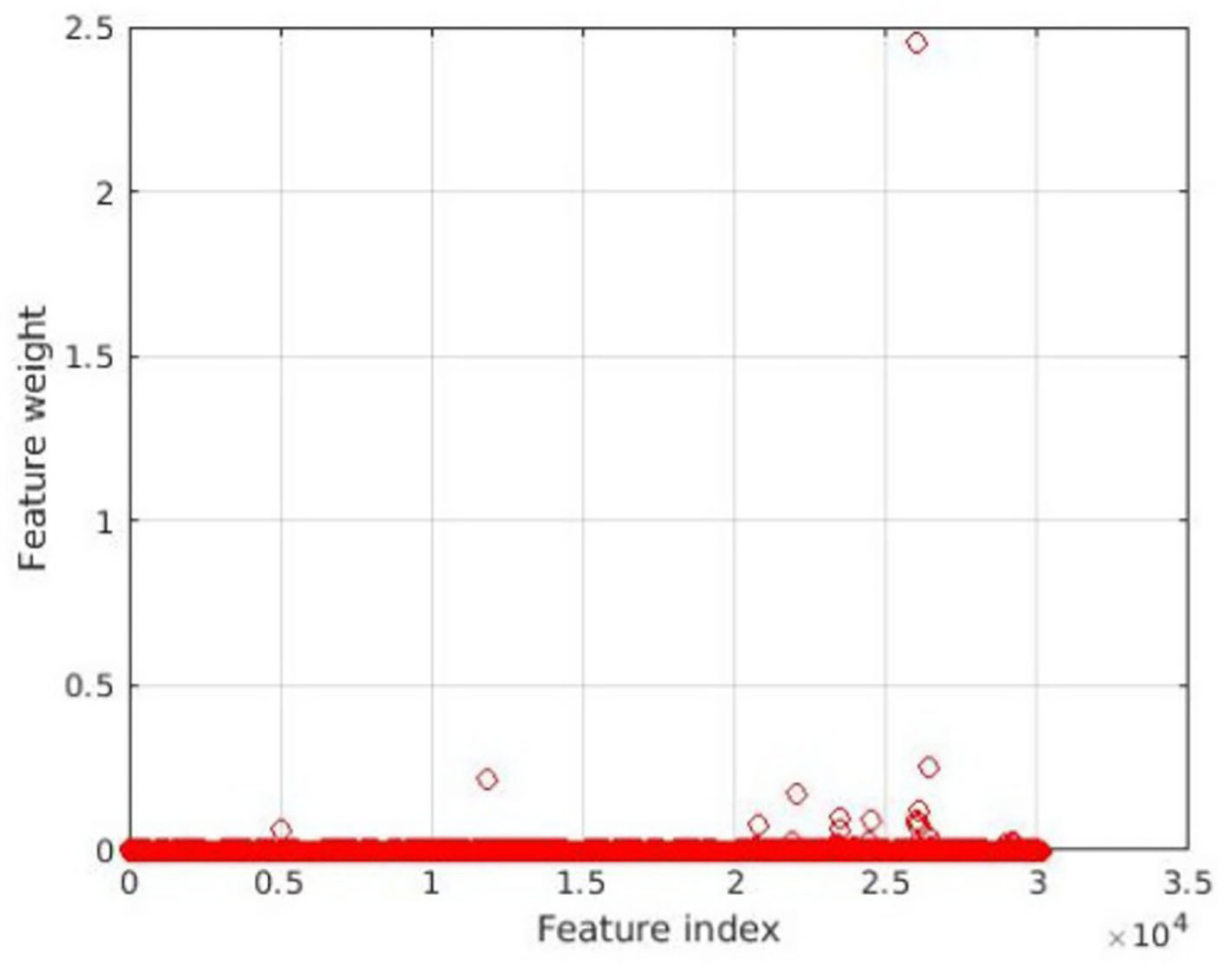
Feature selection through neighborhood component analysis.

**Figure 2:**
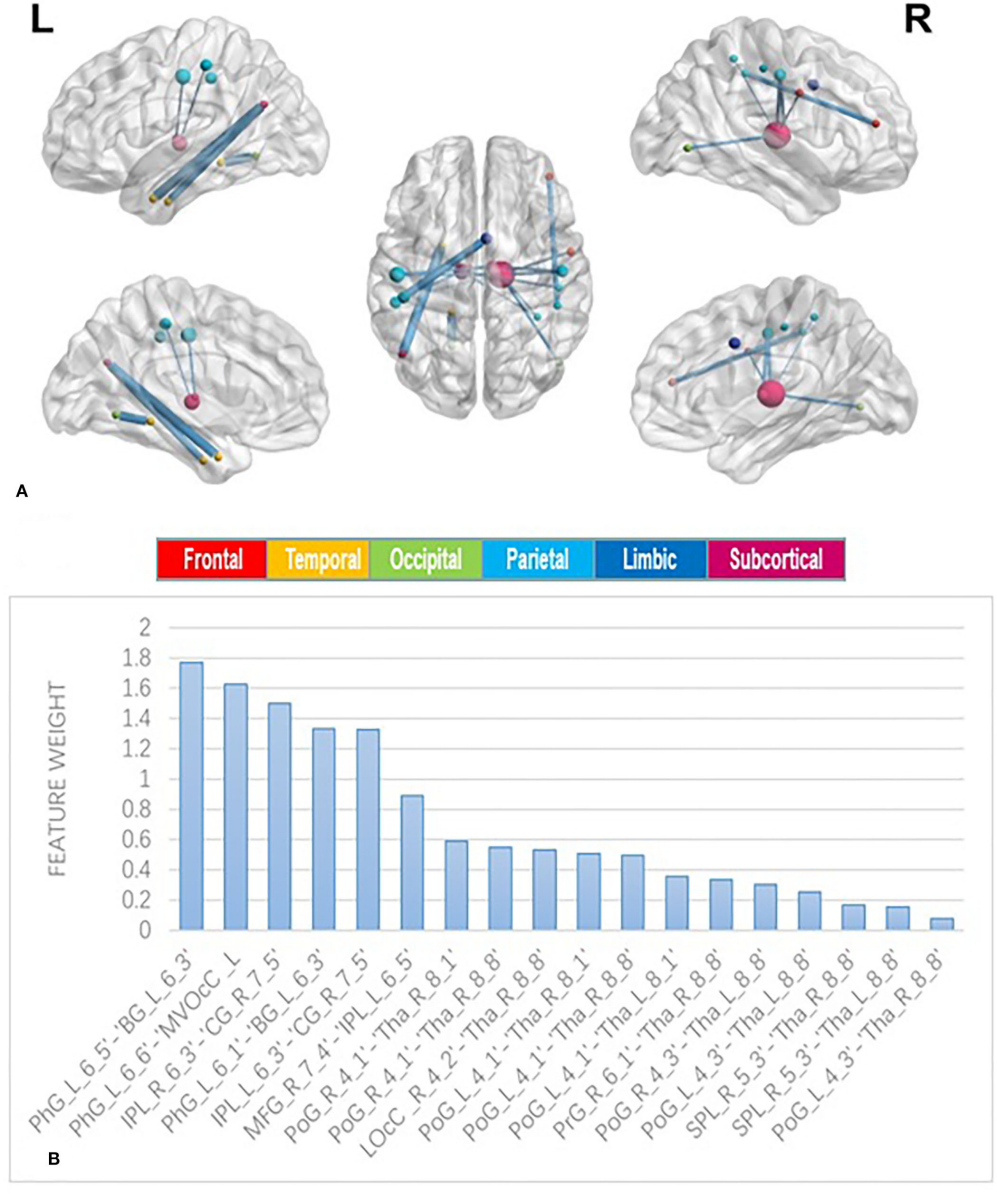
Edges used as features in the classification procedure. **(A)** depicts the location of the 18 edges most consistently selected as relevant features to discriminate patients with methamphetamine use disorder (MUD) from healthy controls (HCs). Brain nodes are scaled according to the number of edges connected to them. Edges are scaled according to the weight value. **(B)** shows the rank of importance of each feature in identifying MUD in the linear support vector machine (SVM) classifier. MFG, middle frontal gyrus; PrG, precentral gyrus; PhG, parahippocampal gyrus; SPL, superior parietal lobule; IPL, inferior parietal lobule; PoG, postcentral gyrus; Tha, thalamus; BG, basal ganglia; MVOcC, medioventral occipital cortex; LOcC, lateral occipital cortex; CG, cingulate gyrus.

The feature selection results using NCA are shown in Figure 1; 18 features assigned with non-zero weights were screened out and then fed into a linear SVM. For linear SVM, a key advantage is that the importance of each feature is directly related to its weighted coefficient, which enabled us to identify the most powerful discriminative features. As shown in Figure 2, the Figure 2A depicted the location of the discriminative features, such as 21 brain regions and 18 edges, Figure 2B showed the rank of importance of each feature in identifying MUD in the linear SVM classifier. The top 6 discriminative features were mainly involved the key nodes of default mode network (DMN) subsystems. All the remaining 12 discriminative features were related to the thalamic connections within the cortico-striato-thalamo-cortical (CSTC) loop. See Figure 2 for more details.

### Classification performance

**Figure 3:**
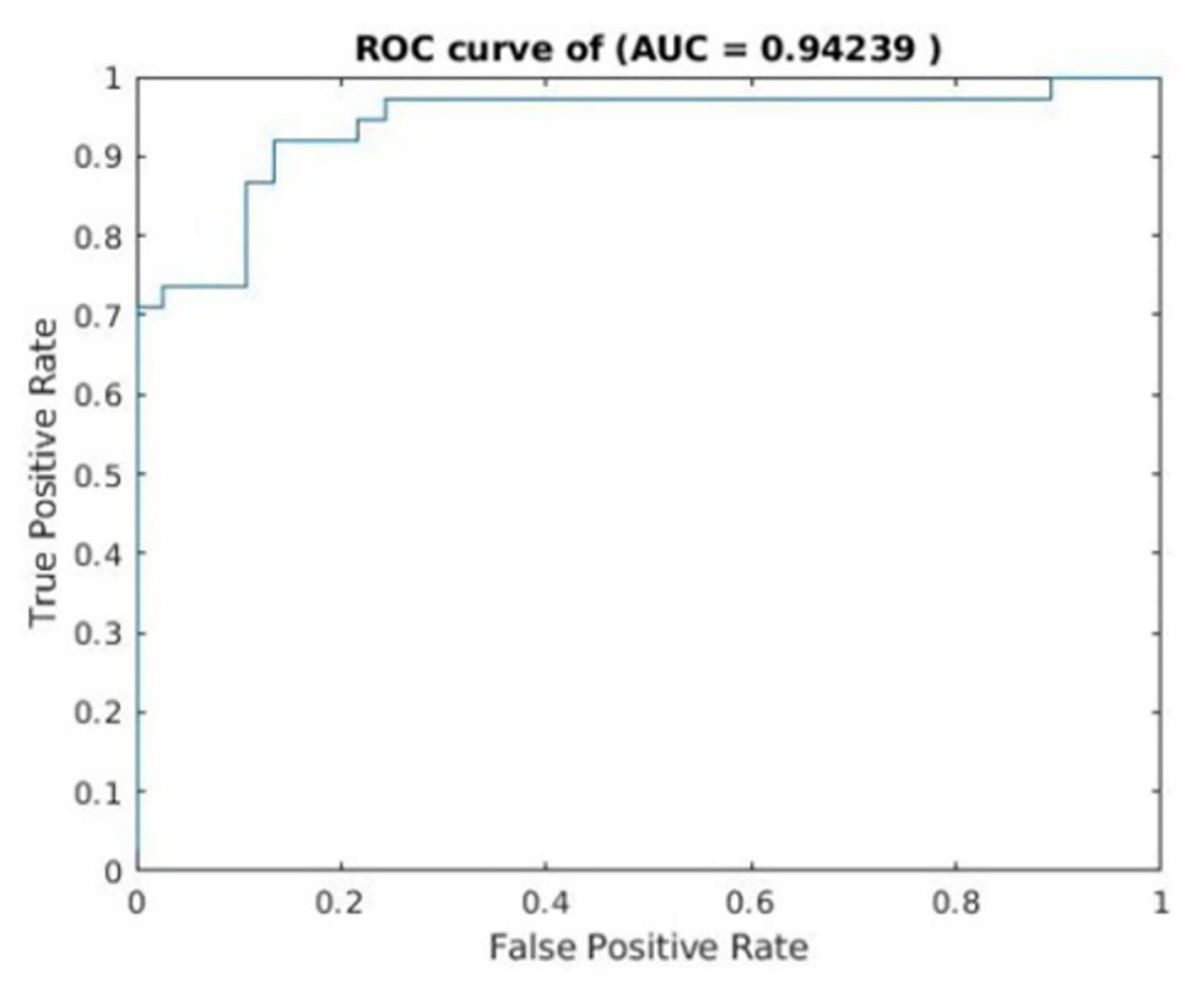
Receiver operating characteristics curves for cross-validated prediction performance of classifiers trained on resting-state functional magnetic resonance imaging (rs-fMRI) data.

The classification accuracy was as high as 88%. The sensitivity and specificity were 86.84 and 89.19%, respectively (Table 2). See Figure 3 for the area under the receiver operating characteristic curve (*AUC* = 0.94) for the corresponding classifier.

**Table 2:** Prediction performance of support vector machine (SVM) classifier trained on resting state functional magnetic resonance imaging (rs-fMRI) data.

**MUD (HCs)**	**Accuracy**	**Sensitivity**	**Specificity**
36 (37)	88.00%	86.84%	89.19%

MUD, methamphetamine use disorder; HCs, Health controls.

### Relationship between discriminative features and MA measures

**Figure 4:**
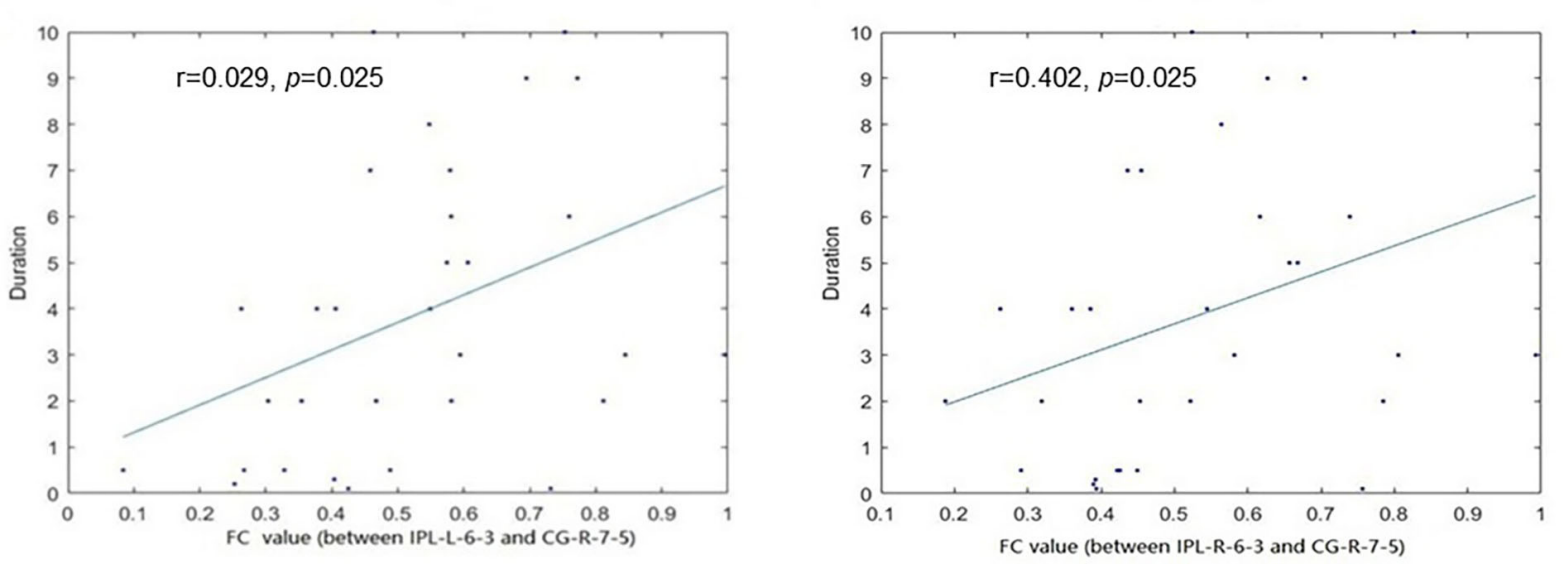
The functional connectivity (FC) between the bilateral inferior parietal lobule (IPL) and right Cingulate gyrus (CG) was significantly correlated with the duration of MA use.

We performed Spearman correlation analyses between the discriminative features and MA measures. The duration of MA use was significantly positively correlated with the FC between the right inferior parietal lobule (IPL) and right cingulate gyrus (CG) (*r* = 0.029, *p* = 0.025), and FC between the left IPL and right CG (*r* = 0.402, *p* = 0.025) (Figure 4). Nevertheless, no significant correlations were found between discriminative features and other MA measures except duration.

## Discussion

This study used an SVM-based ML model of FC data to distinguish individuals with MUD from healthy controls. The classifier achieved very good classification performance, which was close or superior to that in previous studies using data of other modes, such as task-state fMRI (Gowin et al., 2019), arterial spin labeling (Li et al., 2019), differentially expressed genes (Breen et al., 2016), and heart rate extracted from MA-induced electrocardiogram (Wang et al., 2018). Our findings showed that classifiers based on FC measures were predictive of group membership, indicating that the FC findings might be promising biomarkers for MA-related diagnosis at the individual level. The performance of our model was also superior to that in a previous study using FC data (Yan et al., 2021). In addition to the differences in feature extraction methods, the increment in classification accuracy might also be caused by the different parcellation methods. The novelty of the current study was the selection of the human brainnetome atlas to build the network. Previous studies indicated that compared with voxel-wise and atlas-based parcellation methods, this set of 246 subregions was shown to have represented information more accurately in the network (Paxinos, 2016). It was also worth noting that the present study used the NCA strategy as a feature selection method, which has been successfully applied in some previous studies and proved to improve classification performance (Jin and Deng, 2018; Eryilmaz et al., 2020).

The discriminative features extracted from this classifier are considered as biomarkers to guide disease-related interpretations, which may reveal the patterns of FC alteration in MUD patients and facilitate the elucidation of the neuropathological mechanisms underlying MUD. Remarkably, in the present study, the top 6 discriminative features were mainly involved the key nodes of DMN subsystems, such as precentral gyrus (PrG), middle frontal gyrus (MFG), IPL, and CG, which are highly integrated and synergistically activated in most self-generated experiences (Andrews-Hanna, 2012). Altered DMN function is associated with emotional dysregulation, rumination, and compromised cognitive functions (Hahn et al., 2011; Whitfield-Gabrieli and Ford, 2012; Leech and Sharp, 2014). It also strongly interacts with subcortical areas (such as basal ganglia, BG) and other networks (such as medioventral occipital cortex, MVOcC, a key node in the visual network) (Spreng et al., 2013; Raichle, 2015; Wang et al., 2016), affecting functions such as emotion, cognition, attention, and impulsivity (Fox et al., 2005; Shannon et al., 2011). There is a growing body of evidence that abnormal DMN function and disruptive interactions between DMN and other networks can impair the affective and cognitive processes, resulting in drug craving and relapse (He et al., 2018; Zhang and Volkow, 2019). Our results provided further evidence that the DMN function in patients with MUD needs more attention.

Meanwhile, we found that the remaining discriminative features were associated with the thalamic connections within the CSTC loop underlying both motivated and reward behaviors (Haber and Knutson, 2010). Stimulants including MA can affect the normal connectivity of the CSTC loop, which deemed to be associated with the behavioral effects of stimulants (Jentsch and Taylor, 1999; Goldstein and Volkow, 2011). Within the CSTC loop, the thalamus not only acts as a crucial “relay station” but also plays a key role in the integration of thoughts, executive function, and motor function (Sherman and Guillery, 2001; De Bourbon-Teles et al., 2014; Huda et al., 2019); it might also play a significant role in reward processing and goal-directed behaviors in addiction (Corbit et al., 2003; Huang et al., 2018). Both structural and functional changes of the thalamus along with findings of lower gray matter volumes (Morales et al., 2015), reduced white matter integrity (Li et al., 2017), lower metabolism (Volkow et al., 2001), and altered resting state FC (Liu et al., 2020; Mansoory et al., 2020) have been reported in individuals with MUD. Our findings further supported the importance of CSTC, especially the thalamus, in addiction.

In addition, we found that the FC between the IPL and CG was significantly positively correlated with the duration of MA use. The IPL is involved in visuo-spatial attention and recollective aspects of episodic memory (Corbetta et al., 2000; Wheeler and Buckner, 2004; Davidson et al., 2008; Seghier, 2013; Zhang and Li, 2014). The CG, especially its posterior part, is involved in visuospatial attention (Vogt et al., 1992; Grön et al., 2000) and arousal by a stimuli (Maddock, 1999); it might help predict subsequent relapse in substance users (Kosten et al., 2006). Thus, we speculated that the FC between the IPL and CG might be at least partially affect the decision-making process by directing visuo-spatial attention to the internal world and attributing personal relevance to the retrieved episodic memory. A recent study also yielded a similar result, showing that young binge drinkers were associated with higher FC between the IPL and posterior CG compared with controls (Correas et al., 2016), which might reflect the effect of their previous drug use experiences.

Several limitations are worth noting in the present study. First, the sample size might be limited due to the challenges in recruiting individuals with MUD. In future studies, the model can be trained with a larger cohort and validated with an external sample that has not been used in any training iteration. Second, as almost all patients in the rehabilitation center were male, we were unable to make comparisons between genders, which might lead to a gender bias. Third, only rs-fMRI modality was used in this study; thus, multimodal neuroimaging data are needed in future works to investigate whether they can help achieve a superior predictive power. Finally, a longitudinal study should be taken into consideration to assess the prediction of treatment response in patients with MUD.

## Conclusion

In summary, the present study showed the potential of combining FC data with SVM-based techniques to distinguish MUD patients from healthy subjects. With the identification of the most discriminative features, we hope to improve our understanding of MUD-related neuropathology.

## Data availability statement

The raw data supporting the conclusions of this article will be made available by the authors, without undue reservation.

## Ethics statement

This study was approved by the Ethics Committee of the Second Xiangya Hospital of Central South University (No. S095, 2013). The patients/participants provided their written informed consent to participate in this study.

## Author contributions

The study was conceived and designed by TL and YL. Data were collected by YZ, YH, ML, and YW, and were processed and analyzed by YS. The results were interpreted by YZ, YM, and WY. The manuscript was drafted by YZ, and was revised by JT, QWu, SC, QWa, and YL. All authors listed have made a substantial and direct contribution to the present work and approved the final manuscript to be published.

## Funding

The study was supported by the National Key R&D Program of China (2017YFC1310400 to TL), the Health and Family Planning Commission of Hunan Province Project (B20180484 to YZ), the Provincial Natural Science Foundation of Hunan (2020JJ4795 to TL), and the Science and Technology Bureau, Changsha Project (kq2004106 to YZ).

## Conflict of interest

The authors declare that the research was conducted in the absence of any commercial or financial relationships that could be construed as a potential conflict of interest. The handling editor JY declared a shared affiliation with the authors YZ, YM, QWu, SC, QWa, YH, YW, ML, and TL at the time of review.

## Publisher's note

All claims expressed in this article are solely those of the authors and do not necessarily represent those of their affiliated organizations, or those of the publisher, the editors and the reviewers. Any product that may be evaluated in this article, or claim that may be made by its manufacturer, is not guaranteed or endorsed by the publisher.
